# Comparison of Two Methods of Teaching Hypertension in Under Graduate Medical Students: “Planned Lecture” Versus “Cooperative Learning”

**DOI:** 10.5812/numonthly.4103

**Published:** 2012-03-01

**Authors:** Zahra Sobhani, Farokhlagha Ahmadi, Mohammad Jalili, Zinat Nadia Hatmi, Orkideh Olang, Khadijeh Eslami, Seyed Mansoor Gatmiri

**Affiliations:** 1 Iranian Tissue Bank, Tehran University of Medical Sciences, Tehran, IR Iran; 2 Nephrology Research Center, Tehran University of Medical Sciences, Tehran, IR Iran; 3 Educational Research in Medical Sciences (CERMS), Tehran University of Medical Sciences, Tehran, IR Iran; 4 Department of Social Medicine, Tehran University of Medical Sciences, Tehran, IR Iran; 5 Urology and Nephrology Research Center (UNRC), Shahid Beheshti University of Medical Sciences, Tehran, IR Iran; 6 Hamedan University of Medical Sciences, Hamedan, IR Iran

**Keywords:** Hypertension, Teaching Methods, Lecture, Cooperative Behavior

## Abstract

**Background:**

The direct and indirect negative impacts of hypertension on mortality and morbidity and the deficiencies in physicians’ knowledge on its management prompted us to search for new methods of training this item.

**Objectives:**

In this study, 2 methods of teaching—planned lecture and cooperation—were compared in instructing hypertension to medical students.

**Materials and Methods:**

This study was designed to be a prospective analysis of the efficacy of 2 models of cooperation and planned lecture teaching of hypertension. The medical students, in the second term of the 2010 academic year who were introduced to the nephrology ward for their internal medicine course, were randomly assigned to 2 groups to be taught hypertension by 2 models of cooperation and planned lecture to compare their advantages and disadvantages. In their final exam 2 questions concerning the management of hypertension were asked with regard to evaluating the long-term impact of the models on learning. Data were analyzed by paired t-test to compare pre- and post-test in each group, and independent t-test was used to compare the average and standard deviation scores between groups.

**Results:**

Fifty-one students participated in the study. The total number of students in the lecture (group 1) and cooperation (group 2) methods was 28 and 23, respectively. By independent t-test, differences in test scores indicated a similar achievement of the 2 methods for the endpoint of basic knowledge (P = 0.253). But, the cooperation method was more successful in transferring abilities, primarily in the areas of workup and treatment (P < 0.05).

**Conclusions:**

The study findings show that both methods can set in the optimal training for hypertension to students but that the cooperative method is more effective for deduction analysis.

## 1. Background

The WHO announced that the global burden of noncommunicable diseases continues to grow and dealing with it appears to be one of the major challenges for development and growth in the 21st century—responsible for 60% of all deaths globally, with 80% of deaths due to non-communicable diseases occurring in low- and middle-income countries (1). The care of patients is resource-intense, and most societies must cope with increasing demand for health resources, forcing them to make choices about the provision of health services. This situation, as discussed, is more prominent in low- and middle-income countries, where the allocated financial resources do not reflect the rates of increase in demand for the service.

A significant increase in the prevalence of hypertension from 1988 to 2004 was also shown in the US by Ostchega et al., who emphasized on the fact that its control in certain subgroups may be challenging (2). The prevalence of hypertension in the first national health surveys for the surveillance of risk factors of non-communicable diseases in Iran in 2005 in an 89,000-sample population, was 25.2% (95% CI = 24.4-28.9) in those aged 25-64 years (3), and in the second survey of approximately 30,000 adults aged between 15 and 64 years in 2006, it was 17.4%, of whom one-third was under treatment—only one-third of whom had their hypertension controlled (4). The prevalence of hypertension in rural Shandong Province, China, in 2007 in more than 16,000 persons was found to be 43.8%. Of the 22% of those under treatment, only 3.9% achieved BP control (< 140/90 mm Hg) (5). These findings underscore the necessity of comprehensive integrated strategies to improve approaches.

On the other hand, Hadi et al. found that only 18% of medical interns had acceptable knowledge of hypertension in Shiraz (6). Al-Azzam’s study on physicians’ knowledge of the treatment of hypertension in Jordan showed that the practices of new graduates from medical school were not better than those of older graduates and that most undertreat hypertension (7). Some believe that the lack of physicians’ knowledge on hypertension may be due to the teaching methods and as time passes, the renewal of scientific information becomes slower. Educational interventions appear to be mandatory (8-10). On one hand, medicine is an ever changing science; thus, we need to teach students to be lifelong learners. On the other hand, the management of patients requires skills that are unrelated to pure knowledge. Carter showed a non-significant trend between knowledge and BP control, (odds ratio = 0.84; P = 0.130) (11). It appears that different teaching techniques may be necessary to improve various elements of clinical skills. If we invest in methods to make education stimulating and challenging but pleasant, the utilization of resources would be appropriate to achieve better outcomes for patients.

Although there are many ways to impart knowledge, the most optimal method (lecture, simulator practice, cooperative learning and even traditional lecturing) has yet to be determined. Lecturing large groups is one of the oldest forms of teaching. Alternatively, there is another well-known educational technique known as cooperative learning, in which students in small groups collaborate to improve their learning capacity as well as that of others who are in their group. Theoretical support for this method is based on the social interdependence, cognitive-developmental, and behavioral-learning theories (12).

## 2. Objectives 

The direct and indirect negative impact of hypertension on mortality and morbidity and the defect in physicians’ knowledge on its management have prompted us to search for new methods of training this item. In this study, 2 methods of teaching to medical students, planned lecture and cooperation, were compared on the topic of hypertension.

## 3. Materials and Methods

Routinely, medical students in the 4th to 5th year of education are introduced as separate groups to the nephrology ward for 2 weeks in their internal medicine course in each term of the academic year, and in their curriculum, hypertension is one the topics that should be covered. At the end of the internal medicine course, they should pass an exam with multiple choice questions (MCQs). In the present study, groups in the second term of the 2010 academic year who were introduced to the ward were randomly assigned to be taught hypertension in 2 models of cooperation and planned lecture to compare their advantages and disadvantages. On the final exam, 2 questions concerning the management of hypertension were asked to evaluate the long-term impact of the models on learning.

The session for each group started with an explanation of the project and the significance of their feedback for the department to make formal changes in the method of education. Then, they responded to a questionnaire with 30 questions (MCQs, open questions, and fill-in-the-blank areas) before and after the teaching session on 3 areas of the theoretical fundamentals of disease, patient workup, and management. Finally, the data were analyzed using SPSS, version 11.5. Data were analyzed by paired t-test to compare pre and post-test performance in each group, and independent t-test was used to compare the average and standard deviation scores between groups.

## 4. Results 

Fifty-one students participated in the study. The total number of students in the lecture (group 1) and cooperation (group 2) methods was 28 and 23, respectively. Paired sample t-test was conducted to evaluate the impact of teaching on the students’ scores in the area of basic knowledge, treatment, and workup of hypertension by pre- and post-test. There was a statistically significant increase in post-test scores in both groups. The Eta squared statistic indicated a large effect size (Table 1).

**Table 1:** Comparison of the Impact of Two Methods of Education on the Student Training

	**Pre Test** **Mean ± SD**	**Post Test** **Mean ± SD**	**T (df)**	***P*value**	**Eta Squared**
Basic knowledge					
Group1	6.4 ± 2.4	8.6 ± 1.7	-4.89 (26)	0.000	0.479
Group2	6.7 ± 1.9	8.9 ± 1.1	-4.07 (17)	0.001	0.494
Treatment					
Group1	6.3 ± 2.3	9.0 ± 1.9	-5.55 (26)	0.000	0.542
Group2	6.6 ± 2.5	10.2 ± 1.8	-5.54 (17)	0.000	0.644
Workup					
Group1	5.4 ± 1.5	6.3 ± 0.9	-3.16 (26)	0.004	0.278
Group2	5.6 ± 6.8	6.8 ± 0.4	-3.55 (17)	0.002	0.426

By independent t-test, differences were obtained in test scores, indicating similar efficacies of the methods in basic knowledge (P = 0.253). Differences between mean scores in the 2 groups were mainly in the areas of workup and treatment (P < 0.05) (Table 2). In the last phase of the study, 2 questions from the researchers were included on the exam to compare the impact of education method on their long-term capabilities on that specific subject. According to the results of the exams and the independent sample t-test, the difference was not significant. For these 2 questions, the p values were 0.311 and 0.282, respectively. The responses of students to open questions about their opinion and feedback on the methods of teaching were mainly having the feeling of being respected, feeling more confident, having a better understanding of their future professional responsibilities, and having more motivation for learning more in cooperation method versus the faster and more compact learning by programmed lecturing.

**Table 2:** The Results of Students’ Score Between Group 1 (Lecture) and Group 2 (Cooperation)

	Mean ± SD	T (df)	*P* value	Mean Difference, (95%CI )	Eta Squared
Knowledge		T (43.38) = -1.159	0.253	-0.44 (-1.25-0.36)	0.03
Group1	8.56 ± 1.67				
Group2	9.0 ± 1.00				
Treatment		T (48) = -2.445	0.018	-1.25 (-2.29- -2.22)	0.11
Group1	8.96 ± 1.89				
Group2	10.21 ± 1.70				
Workup		T (34.17) = -2.807	0.008	0.536 (-0.92- -0.15)	0.14
Group1	6.33 ± 0.92				
Group2	6.87 ± 0.34				

## 5. Discussion 

The main aim of medical education is to improve performance and save patients’ lives. Although knowledge acquisition by medical trainees is important, the assessment of their skills, in practice, takes time. No single method of teaching is effective for all groups of learners. It seems that focused, short lectures affect a positive change in the behavior of learners for a special topic, especially in more specialized trainees, such as residents (13). According to the results of this study, both methods were effective techniques in teaching some theoretical basics of the disease, but with regard to management and workup, the cooperation method was more effective. However, another essential step in the learning cycle is the engagement of the learner by motivation and insight into the importance of his future tasks, making him a lifelong learner. The results of this study, consistent with the literature showed, that the cooperation method was more successful than lecturing in achieving these goals (14, 15). Students find such an environment safer to express themselves and make decisions, a value that can not be underestimated.

A meta-analysis that compared the effect of traditional teaching methods on learning with cooperation was investigated in 193 students. The results showed that in more than 50% of cases, education in the cooperative method was more efficient than the lecture method, but in was less efficient in less than 10% of students tested (16). A review of 122 studies comparing the cooperative learning method with the lecture-based learning method has shown that while the cooperative learning method is not always better than the alternative method, students are left with a higher sense of success and that the cooperative learning method rarely has an adverse effect on learning outcomes (17).

Kari’s research found that students who were taught under the cooperative method were more willing to continue learning under this method at the end of the study (18). Comparing 2 teaching methods (team training method and lecturing with answer-questions) in nursing students in Iran showed that the team training method was more effective than the other method (19).

It seems that the cooperative method is more effective and efficient than the lecture method. Also, comprehensive studies are recommended to evaluate the effect of the short-term and long-term impact of the cooperation method on learning through students’ attitudes about working within teams, their sense of professional development, and comfort and satisfaction.
